# Invasive physiologic assessment of coronary artery stenosis by resting full-cycle ratio and fractional flow reserve: a prospective observational study

**DOI:** 10.1038/s41598-023-43082-1

**Published:** 2023-09-22

**Authors:** Oh-Hyun Lee, Ji Woong Roh, Yongcheol Kim, Seok-Jae Heo, Eui Im, Deok-Kyu Cho

**Affiliations:** 1 Division of Cardiology, Department of Internal Medicine, Yonsei University College of Medicine and Cardiovascular Center, Yongin Severance Hospital, Yongin, South Korea; 2 Division of Biostatistics, Department of Biomedical Systems Informatics, Yonsei University College of Medicine, Seoul, South Korea

**Keywords:** Blood flow, Interventional cardiology

## Abstract

Resting full-cycle ratio (RFR), an alternative to fractional flow reserve (FFR) for evaluating intermediate coronary artery stenosis, helps reduce patients’ time, cost, and discomfort. However, the validation data for RFR and FFR are lacking. We aimed to assess the diagnostic accuracy of RFR and FFR and evaluate effective decision-making for revascularization using their values. Patients subjected to an invasive physiological study for intermediate coronary artery stenosis in Yongin Severance hospital between October 2020 and April 2022 were prospectively and consecutively recruited. We evaluated the correlation between RFR and FFR measurements and the diagnostic performance of RFR (≤ 0.89) versus FFR (≤ 0.80). In all, 474 intermediate coronary stenosis lesions from 400 patients were evaluated using RFR and FFR values. There was a strong linear relationship between RFR and FFR (r = 0.75, 95% CI 0.70–0.78, *p* < 0.01). Comparing diagnostic performance between RFR and FFR, RFR demonstrated diagnostic accuracy, sensitivity, specificity, positive predictive value (PPV), and negative predictive value (NPV) of 85.0%, 80.0%, 86.7%, 67.1%, and 92.7%, respectively. We analyzed the RFR value in the hyperemia zone (0.86–0.93) according to positive (RFR: 0.86–0.89) and negative (RFR: 0.90–0.93) areas. PPV in positive area is 47.8% (95% Confidence Interval [CI]: 33.8% to 62.0%) and NPV in negative area is 87.7% (95% CI: 80.3% to 93.1%). Excellent correlation exists between RFR and FFR and the diagnostic value of RFR without hyperemia compared with FFR in establishing the accurate functional significance of coronary artery stenosis was shown. RFR alone could evaluate the functional significance of coronary artery stenosis without unnecessary hyperemia, except in the positive area.

**Trial registration**: URL: http://trialsearch.who.int; Unique identifier: KCT0005255.

## Introduction

Fractional flow reserve (FFR) is a reliable index of the functional severity of coronary artery stenosis, which is determined using coronary pressure measurement during cardiac catheterization. Notably, several studies have shown that invasive coronary physiology assessment could improve clinical outcomes by appropriately selecting patients who may benefit from percutaneous coronary interventions (PCI) with stent implantation^1–3^. Therefore, current guidelines recommend evaluating coronary pressure-derived FFR to evaluate the hemodynamic significance of intermediate coronary lesions in patients with symptomatic angina and undocumented ischemia^4,5^. However, FFR has several disadvantages, including the need to induce maximal hyperemia, which requires additional time and cost and to minimize the effect of coronary microcirculation by administering pharmacological agents such as intravenous or intracoronary adenosine, which causes discomfort for patients^6^. Therefore, the hurdle of hyperemia in clinical practice could reduce FFR use. Recent studies have reported that the rate of FFR-guided PCI for intermediate lesions was only 6.1% in the United States, 8.2% in Japan, and 5.1% in South Korea^7–9^.

Instantaneous wave-free ratio (iFR) has been developed to overcome the limitation of FFR requiring maximal hyperemia. With accumulating evidence, iFR, which is similar to FFR, is also recommended for evaluating the hemodynamic significance of intermediate coronary stenosis in the current guidelines^10–13^. Notably, several non-hyperemic pressure ratios (NHPR) have been developed, including the entire cardiac cycle, and one of them is the resting full-cycle ratio (RFR). RFR evaluates the hemodynamic significance of coronary stenosis by identifying the lowest distal arterial pressure (Pd)/arterial pressure (Pa) ratio within the entire cardiac cycle. The validation of a novel non-hyperemic index of coronary artery stenosis severity i.e., the RFR (VALIDATE RFR) study reported that RFR is diagnostically equivalent to iFR^14^. However, data regarding the comparison between RFR and FFR in patients with intermediate coronary artery disease are limited^15–17^. Therefore, the present study aimed to assess the diagnostic value of RFR and FFR in real-world practice.

## Methods

### Study population

The Invasive physiologic assessment of coronary artery stenosis by RFR and FFR (ICE-FLOWER) study was a prospective, single-center observational study conducted at Yongin Severance Hospital in South Korea. Patients aged > 19 years with suspected ischemic heart disease (IHD) between October 2020 and April 2022 were enrolled in this study if diagnosed with de novo stenosis. We excluded patients with single lesion in acute coronary syndromes, left main coronary artery disease, stenosis in a coronary artery bypass graft, and a life expectancy of < 1 year. For non-culprit lesions in patients with acute coronary syndrome, we performed RFR/FFR immediately if the patient was stable, and staged RFR/FFR measurement was done within 7 days if the patient was unstable. This study protocol was approved by the Institutional Review Board of Yongin Severance Hospital (approval number: 9–2020-0072), and all participants provided written informed consent before participating in the study. The study protocol was registered in the International Clinical Trial Registry Platform (KCT0005255) on July 24th, 2020 (https://trialsearch.who.int/Trial2.aspx?TrialID=KCT0005255) and adhered to the ethical guidelines of the Declaration of Helsinki. The funding sources did not participate in the design or conduct of the study, analysis or interpretation of the data, or the decision to submit the manuscript for publication.

### Cardiac catheterization and quantitative coronary angiography

Coronary angiography (CAG) was performed by five interventional cardiologists with extensive experience in CAG and PCI according to current guidelines and standard technique using a femoral, proximal, or distal radial approach^4,12^. Diameter stenosis percentage, minimal and reference lumen diameter, and lesion length were assessed with quantitative coronary angiography (QCA) using CASS workstation 7.4 (Pie Medical Imaging, Maastricht, Netherlands). All QCA images were retrospectively re-analyzed by two independent observers with > 5 years of experience in cardiac catheterization. All diameters were determined as the average of the diameter values obtained independently. The reference diameter was determined using the proximal and distal reference diameters.

### Coronary physiologic measurements and assessment

Physiological assessments of the intermediate coronary lesions (50%–90% diameter stenosis using QCA analysis) were performed as follows. All coronary physiologic measurements were performed using a 0.014″ intracoronary wireless pressure wire (PressureWire™ X Guidewire [Abbott Vascular Inc., Santa Clara, CA, USA]) and automatically calculated using the QUANTIEN™ system (Abbott Vascular Inc.) and OPTIS™ Mobile System (Abbott Vascular Inc.). The pressure wire was equalized to the aortic pressure, and equalization was then performed after placing the pressure wire on the tip of the guiding catheter and removing contrast media using saline flushing. First, the pressure wire was advanced distally to a target vessel to evaluate the RFR value. The instantaneous Pd/Pa was continuously measured throughout five cardiac cycles to calculate RFR, defined as the point where the Pd/Pa ratio was the lowest during the entire cardiac cycle. After the RFR measurement, the pressure wire was pulled back into the tip of the guiding catheter to check the presence of pressure drift. A final Pd/Pa between 0.97 and 1.03 is considered acceptable^18,19^. After confirming that there was no pressure drift, the pressure wire was re-advanced to the distal portion of the target vessel to evaluate the FFR value. In all lesions, FFR values were measured with hyperemia, achieved using an intracoronary (IC) bolus injection of 2 mg nicorandil (Sigmart®; Chugai Pharmaceutical Co., Ltd., Tokyo, Japan). Based on previous studies regarding IC bolus injection of nicorandil for hyperemia, FFR measurement was obtained during maximal hyperemia, defined as a period between 20 and 50 s after IC bolus injection of nicorandil^6,20^. The pressure drift was checked after FFR measurement. In patients with multivessel disease, there were sufficient time intervals between measurements of each vessel^21^.

### Definitions

We evaluated the correlation between RFR and FFR measurements and the diagnostic performance of RFR to identify FFR-positive coronary stenosis. Hemodynamically significant stenosis was defined as FFR ≤ 0.80 and RFR ≤ 0.89. Based on the actual RFR cut-off of 0.89, the gray zone with an RFR value between 0.86 and 0.93, called the “hyperemia zone,” is further subdivided into the positive (RFR 0.86–0.89) and negative (RFR 0.90–0.93) areas^22^. The diagnostic accuracy, positive predictive value (PPV), and negative predictive value (NPV) of RFR in the hyperemia zone were re-evaluated. We also evaluated the relationship between RFR and Pd/Pa.

### Statistical analysis

All data were expressed as mean ± standard deviation (SD) or numbers (%) of patients. The 95% confidence intervals (CIs) of the means of continuous variables and percentages of categorical variables were calculated with t-tests and Clopper–Pearson (exact) approaches, respectively. Pearson’s correlation coefficient (r) between RFR and FFR was computed with 95% CIs. The receiver operating characteristic (ROC) curve was used to represent the overall prediction performance of the RFR for FFR ≤ 0.80 and Pd/Pa ≤ 0.92 with the area under the ROC curve (AUC). Youden’s index was used to determine the optimal RFR cut-off against FFR ≤ 0.80 and the prediction of Pd/Pa ≤ 0.92. Prediction performance measures, such as sensitivity, specificity, PPV, NPV, and accuracy were calculated. The cut-off value of RFR calculated using Youden’s index was determined to be 0.89, consistent with the value in previous studies^10,14^. Statistical significance was set at *P* < 0.05 (two-sided). All statistical analyses were performed with SPSS statistical software (SPSS version 25.0 for Windows; IBM Corp., Armonk, NY, USA) and R software (version 4.3.0; R Foundation for Statistical Computing, Vienna, Austria).

## Results

### Study population

**Figure 1:**
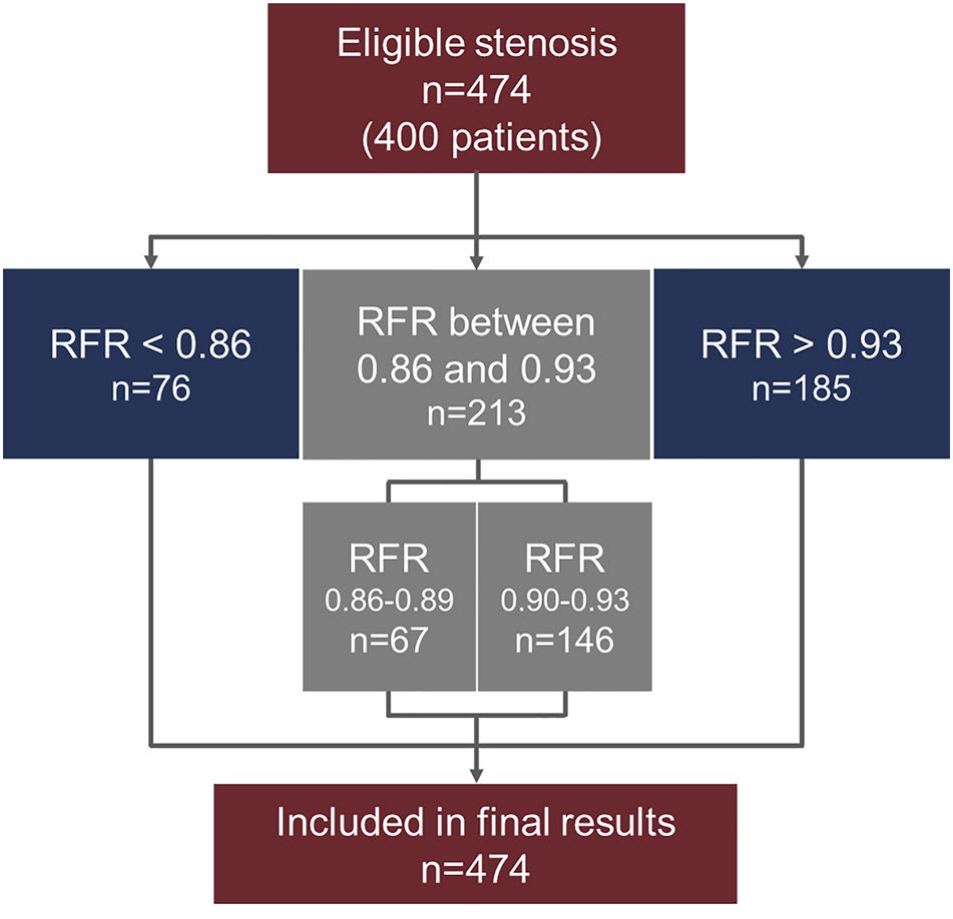
Study flowchart. RFR, resting full-cycle ratio; FFR, fractional flow reserve.

A total of 474 stenoses from 400 patients were investigated and included in this study. A STAndards for the Reporting of Diagnostic Accuracy Studies (STARD)-type flow chart depicts this process as shown in Fig. 1.^23^ Participants’ baseline clinical and angiographic characteristics are shown in Tables 1 and 2. The overall mean age was 66.2 ± 10.9 years, and 68.6% of patients were male. The common clinical presentation was stable angina (73.9%), followed by unstable angina (16.5%), and the lesions were located most often in the left anterior descending artery (63.5%). The distribution of the RFR and FFR values is shown in Supplementary Fig. S1 online. The study population comprised patients with angiographically intermediate stenosis (diameter stenosis [%]: 59.6 ± 6.1 using QCA). The mean ± SD and median values with interquartile range (IQR) of RFR were 0.91 ± 0.08 and 0.92 (IQR: 0.88–0.96), respectively. Notably, 120 (30.2%) vessels had RFR ≤ 0.89 (see Supplementary Fig. S2).

**Table 1:** Baseline clinical characteristics of the study population (N = 400).

Characteristics	Mean ± SD or n (%)	95% CI*
Age, years	66.2 ± 10.9	65.2–67.3
Male sex	273 (68.6)	63.8–73.1
Height, cm	162.3 ± 8.9	161.4–163.1
Weight, kg	67.3 ± 11.6	66.1–68.4
Body mass index, kg/m^2^	25.5 ± 3.4	25.1–25.8
Hypertension	278 (69.8)	65.1–74.3
Diabetes mellitus	188 (47.2)	42.2–52.3
Dyslipidemia	369 (92.7)	89.7–95.1
Current smoking	65 (16.3)	12.8–20.3
Prior PCI	79 (19.8)	16.0–24.1
Prior myocardial infarction	40 (10.1)	7.3–13.4
Prior cerebrovascular accident	24 (6.0)	3.9–8.8
Prior coronary bypass graft	2 (0.5)	0.1–1.8
Peripheral artery disease	10 (2.5)	1.2–4.6
Congestive heart failure	47 (11.8)	8.8–15.4
Atrial fibrillation	17 (4.3)	2.5–6.8
Chronic kidney disease, ≥ stage 3	57 (14.3)	11.0–18.2
Dialysis	5 (1.3)	0.4–2.9
LVEF, %	58.3 ± 10.6	57.2–59.3
Average E/e’	10.6 ± 4.6	10.1–11.1
Systolic blood pressure, mmHg	142.0 ± 25.4	139.5–144.5
Diastolic blood pressure, mmHg	72.0 ± 12.4	70.8–73.2
Heart rate, bpm	71.6 ± 12.0	70.4–72.8
Clinical presentation		
Stable angina	294 (73.9)	69.3–78.1
Unstable angina	66 (16.5)	13.1–20.6
NSTEMI	31 (7.8)	5.3–10.8
STEMI	7 (1.8)	0.7–3.6
RFR/FFR measurement for non-culprit lesion in AMI during index procedure	35 (8.8)	4.9–12.0
Laboratory findings		
Total cholesterol, mg/dL	156.1 ± 49.7	151.1–161.1
Triglyceride, mg/dL	160.2 ± 228.8	136.4–184.1
HDL-cholesterol, mg/dL	48.1 ± 12.5	46.8–49.4
LDL-cholesterol, mg/dL	96.0 ± 40.9	91.7–100.3
Creatinine, mg/dL	1.0 ± 0.9	0.9–1.1
Hemoglobin, g/dL	13.8 ± 1.9	13.6–14.0
Platelet count, 10^3^/μL	230.4 ± 60.5	224.4–236.3
CRP, mg/L	5.1 ± 19.4	2.9–7.2
Pre-procedural medication		
Aspirin	354 (88.9)	85.4–91.9
P2Y_12_ inhibitor	367 (92.2)	89.1–94.6
Clopidogrel	319 (80.2)	75.9–84.0
Ticagrelor	45 (11.3)	8.4–14.8
Prasugrel	1 (0.3)	0.0–1.4
Oral anticoagulation	23 (5.8)	3.7–8.5
ACEi or ARB	180 (45.2)	40.3–50.3
Beta-blocker	121 (30.4)	25.9–35.2
Calcium channel blocker	164 (41.2)	36.3–46.2
Statin	352 (88.4)	84.9–91.4

*95% confidence intervals (CI) of the mean.

Abbreviations: SD, standard deviation; PCI, percutaneous coronary intervention; LVEF, left ventricular ejection fraction; NSTEMI, non-ST-segment elevation myocardial infarction; STEMI, ST-segment elevation myocardial infarction; HDL, high density lipoprotein; LDL, low density lipoprotein; CRP, C-reactive protein; ACEi, angiotensin converting enzyme inhibitor; AMI, acute myocardial infarction; ARB, angiotensin receptor blocker.

**Table 2:** General Characteristics of Epicardial Stenosis (N = 474).

Characteristics	Mean ± SD or n (%)	95% CI*
Vessel		
Left anterior descending artery	301 (63.5)	59.0–67.8
Left circumflex	69 (14.6)	11.5–18.1
Right coronary artery	104 (21.9)	18.3–25.9
Lesion location		
Proximal	181 (38.2)	33.8–42.7
Mid or distal lesion	282 (59.5)	54.9–63.9
Diffuse lesion	11 (2.3)	1.2–4.1
Stenosis characteristics		
Lesion length, mm	12.2 ± 6.2	11.6–12.8
Reference vessel diameter, mm	2.8 ± 0.7	2.7–2.8
Percentage of diameter stenosis	59.6 ± 6.1	59.1–60.2
ACC/AHA B2/C Lesion	339 (71.5)	67.2–75.5

*95% confidence intervals (CI) of the mean.

Abbreviations: SD, standard deviation; ACC, American College of Cardiology; AHA, American Heart Association.

### Relationship between RFR and FFR or Pd/Pa

**Figure 2:**
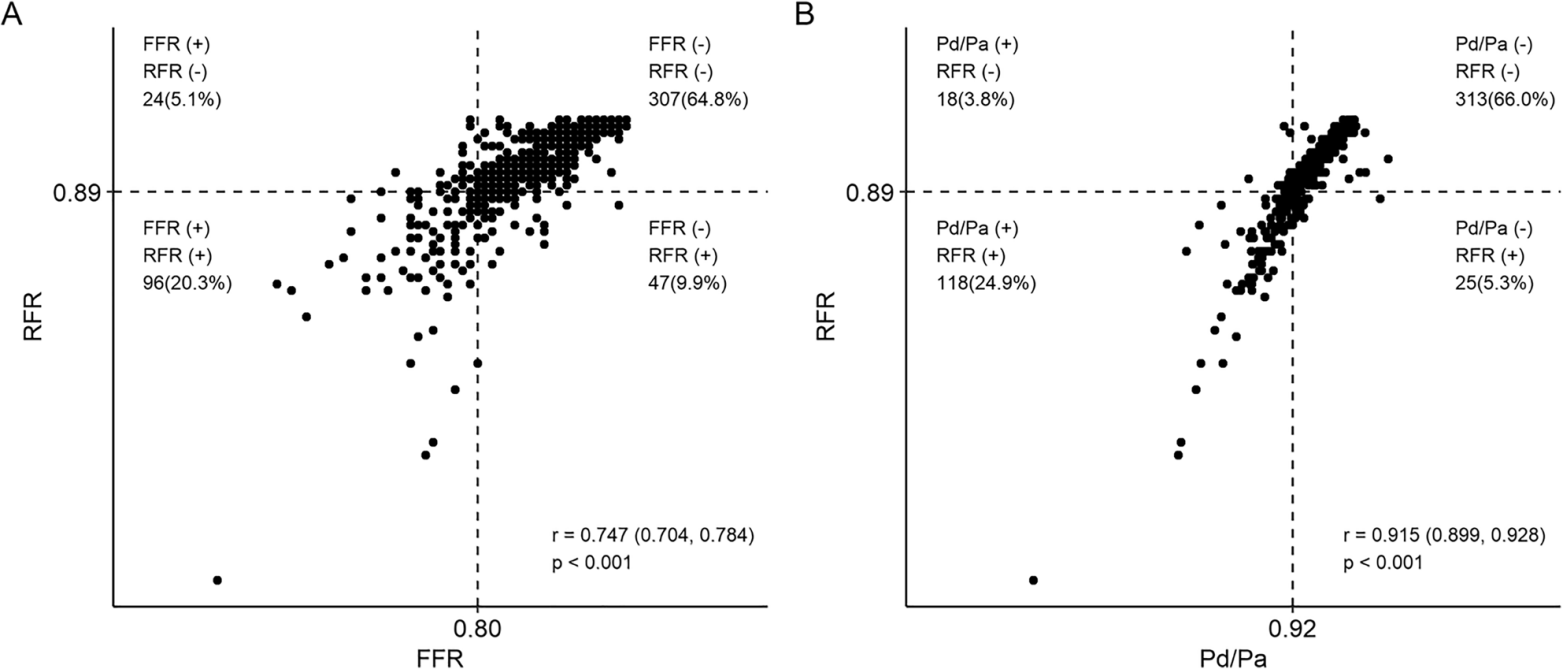
Concordance and discordance among resting full-cycle ratio (RFR) and fractional flow reserve (FFR) or distal coronary pressure (Pd)/ aortic pressure (Pa). (**A**) FFR and RFR showed a significant correlation (r = 0.747; *p* < 0.001), and (**B**) The correlation between RFR and Pd/Pa was also significant, with a similar correlation coefficient (r = 0.915; *p* < 0.001). FFR, fractional flow reserve; RFR, resting full-cycle ratio; Pd, distal coronary pressure; Pa, aortic pressure.

Figure 2A shows the scatterplot of the relationship between RFR and FFR. A strong correlation was noted between both indices (r = 0.75, 95% CI 0.70–0.78, *p* < 0.01). Figure 2B also shows a strong correlation between RFR and Pd/Pa (r = 0.92, 95% CI 0.90–0.93, *p* < 0.01).

**Figure 3:**
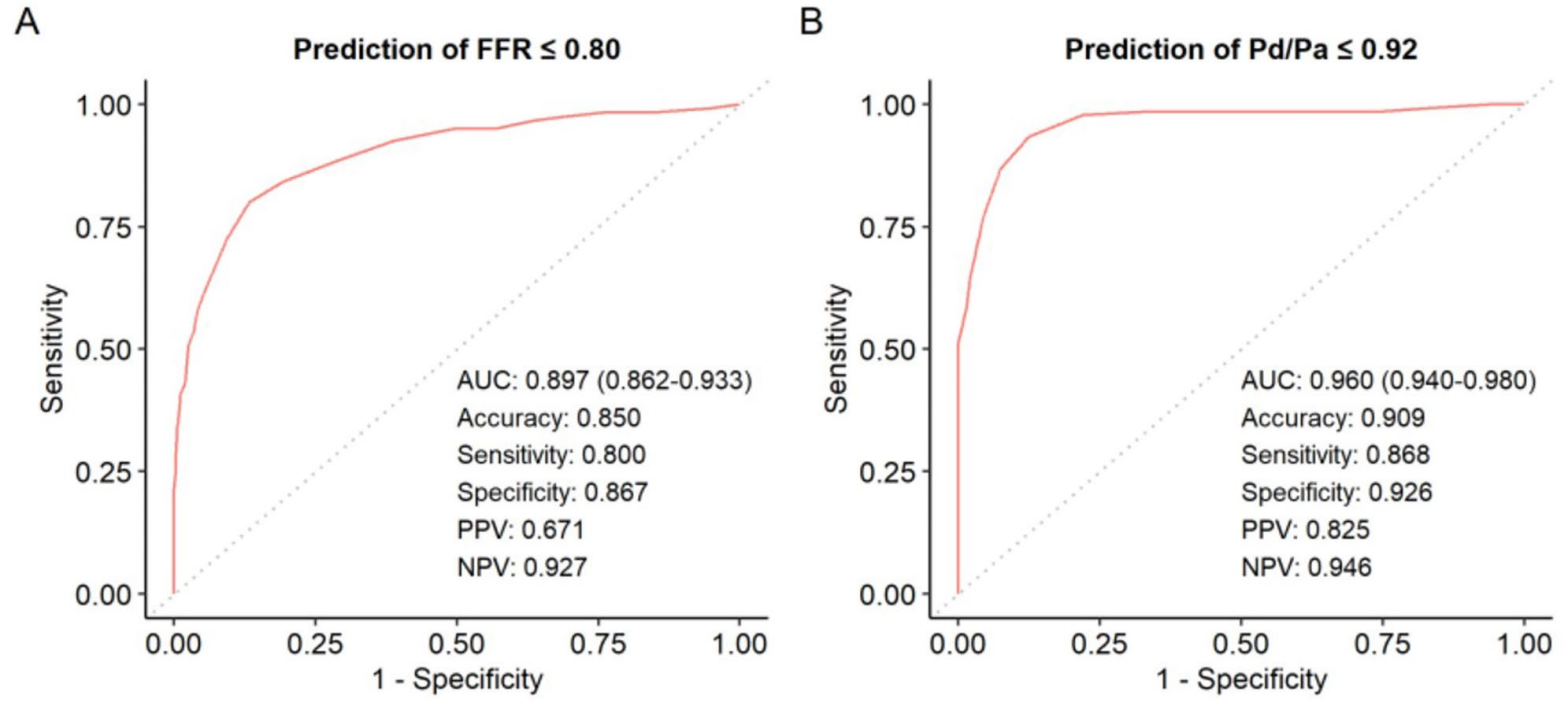
Receiver operating characteristic (ROC) curves. (**A**) ROC curves for RFR versus FFR ≤ 0.80 showed an area under the ROC curve (AUC) of 0.897 (0.862–0.933) and the following values: diagnostic accuracy, 0.85; sensitivity, 0.80; specificity, 0.87; positive predictive value, 0.67; negative predictive value, 0.93. (**B**) ROC curves for RFR versus Pd/Pa ≤ 0.92 showed an AUC of 0.960 (0.940–0.980) and the following values: diagnostic accuracy, 0.91; sensitivity, 0.87; specificity, 0.93; positive predictive value, 0.83; negative predictive value, 0.95. ROC, receiver operating characteristic curve; RFR, resting full-cycle ratio; FFR, fractional flow reserve; Pd, distal coronary pressure; Pa, aortic pressure.

Compared with FFR, RFR demonstrated a diagnostic accuracy of 85.0%. RFR ≤ 0.89 demonstrated a sensitivity, specificity, PPV, and NPV of 80.0%, 86.7%, 67.1%, and 92.7%, respectively in predicting significant FFR ≤ 0.80. ROC analyses for the prediction of FFR ≤ 0.80 showed an AUC (C statistic) of 0.90 (95% CI 0.86–0.93, *p* < 0.01) (Fig. 3A). The optimal cut-off point of RFR was 0.89, with a Youden index of 0.67.

ROC analyses for the prediction of Pd/Pa ≤ 0.92 showed an AUC (C statistic) of 0.96 (95% CI 0.94–0.98, *p* < 0.01). Using Pd/Pa ≤ 0.92 as a reference, the sensitivity, specificity, PPV, NPV, and overall diagnostic accuracy of RFR were determined as 86.8%, 92.6%, 82.5%, 94.6%, and 90.9%, respectively. (Fig. 3b).

### Accuracy of RFR with FFR in the RFR hyperemia zone (RFR: 0.86–0.93)

**Figure 4:**
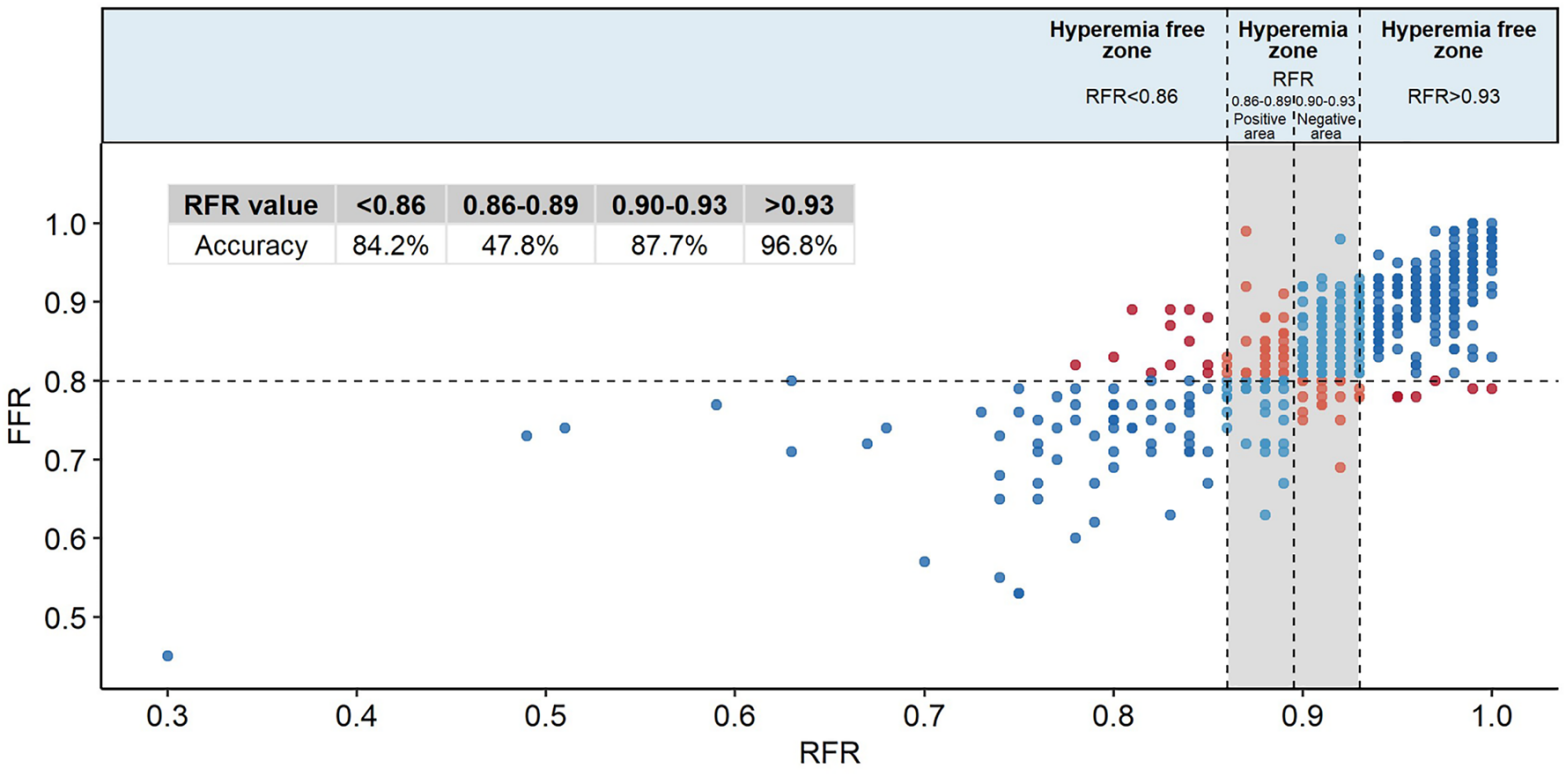
FFR versus RFR in the hyperemia and hyperemia-free zones. Red and blue dots represent the disagreement and agreement, respectively, between the two strategies (RFR vs. FFR) in the hyperemia-free zone (RFR value < 0.86 and > 0.93). Light red and blue dots represent the hyperemia zone (RFR: 0.86–0.93), reclassified into positive (RFR: 0.86–0.89) and negative (RFR: 0.90–0.93) areas.

**Figure 5:**
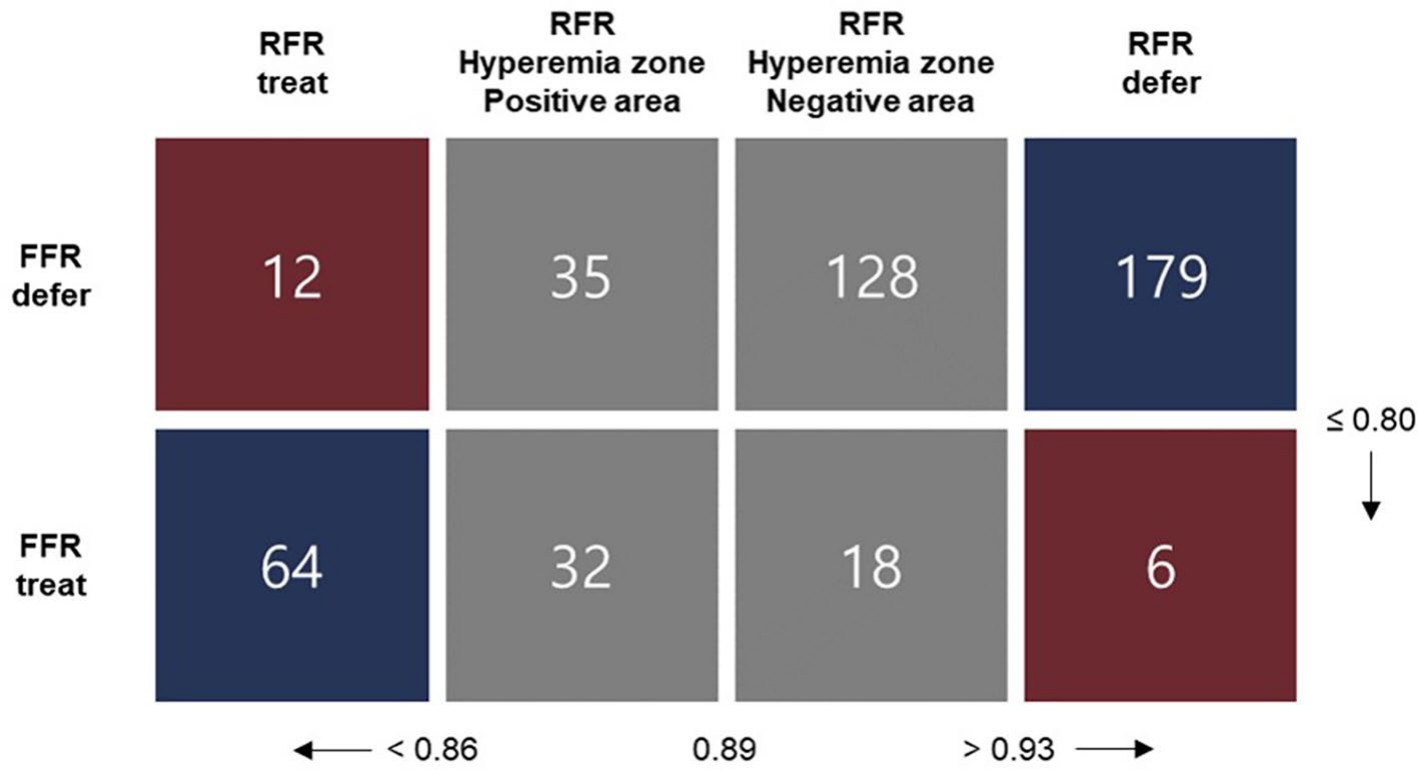
Comparison of patient numbers according to RFR and FFR values in the hyperemia and hyperemia-free zones. Red and blue spots represent the disagreement and agreement, respectively, between the two strategies in the hyperemia-free zone. In the hyperemia-free zone, the accuracy rate is 93.1% (243/261).

In the RFR hyperemia zone (RFR: 0.86–0.93), the value was divided into positive (RFR: 0.86 to 0.89) and negative (RFR: 0.90 to 0.93) areas to evaluate the accuracy of RFR with FFR, which was 47.8% (95% CI 33.8%–62.0%) in the positive area and 87.7% (95% CI 80.3%–93.1%) in the negative area. Outside the hyperemia zone, the accuracy of RFR < 0.86 with FFR ≤0.80, and RFR >0.93 with FFR >0.80 were 84.2% (64/76, 95% CI 72.5%–92.4%) and 96.8% (179/185, 95% CI 92.4–99.0%), respectively (Fig. 4). Therefore, the accuracy of RFR with FFR in the entire outer part of the hyperemia zone was 93.1% (95% CI 88.7–96.2) (Fig. 5).

### Clinical predictors of discordance between FFR and RFR

In univariable analysis for discordance predictors, diabetes mellitus and left anterior descending artery lesion were the factors with p value less than 0.1. However, diabetes mellitus [odds ratio (OR); 1.80, 95% confidence interval (CI); 1.07–3.07; *p* = 0.026] was the only significant factor for predicting discordance between FFR and RFR in multivariate analysis. (Supplementary Table 1).

## Discussion

This prospective observational study (ICE-FLOWER) analyzed the validation of a novel non-hyperemic resting physiologic index using RFR for coronary artery intermediate stenosis in patients with IHD. Herein, we reported several clinically important findings to support the effective application of RFR with FFR in daily clinical practice. First, RFR value showed a good correlation with FFR value in the present study as compared to that in the previous study that used iFR, which is the gold standard of NHPR. Second, compared with the FFR (≤ 0.80) value, RFR (≤ 0.89) value in the present study showed a favorable accuracy rate of 85.0%, which was comparable to that of iFR or FFR in other studies. Third, RFR value demonstrated low accuracy (47.8%) in the positive area of the hyperemia zone (0.86–0.89); therefore, additional FFR would be required to evaluate functional coronary stenosis accurately in the positive area.

FFR-guided PCI has shown superior clinical outcomes in randomized trials, and it is recommended as class 1A in current guidelines for intermediate coronary artery stenosis^4,5,24^. However, the rate of FFR-guided PCI for intermediate coronary lesions in real-world practice is very low because of time, cost, and risk of adenosine- or nicorandil-induced maximal hyperemia^7–9^. NHPRs have emerged recently, and among them, iFR demonstrated non-inferiority compared with FFR in two randomized control trials; further it is recommended in the guidelines for class 1A^10,11^. RFR is one of the NHPR indices, and it has been introduced for obtaining the absolute Pd/Pa value of the entire cardiac cycle^14^. The present study demonstrated a good correlation between RFR and FFR and excellent diagnostic accuracy of RFR (> 85%) as compared to FFR in real-world practice. A few recent studies showed that RFR was diagnostically equivalent to iFR and had a comparable value with FFR^16,17,25^. In two recently published studies comparing RFR and FFR, the accuracy rate was 78% in a study from Germany that analyzed 712 lesions and 79% in a study from Spain that analyzed 380 lesions^16,17^. These accuracy rates were similar to those in previous studies comparing iFR and FFR^26,27^. Regarding the validation of RFR, the present study showed a higher accuracy rate than previous studies.

Discordant findings of NHPRs and FFR have shown worse prognosis compared to concordant negative indices in previous trials. In our study, only diabetes mellitus known as microvascular dysfunction, a discordant factor also identified in previous studies^28^, was found to be a significantly influencing factor. Further, there is an ongoing debate with growing interest whether discordant lesions should be revascularized^29^. To overcome such disadvantages and limitations, few studies have suggested a hybrid approach using FFR and NHPR simultaneously during decision-making for revascularization^22^. However, the accuracy of iFR or RFR in the hyperemia-free zone is reported to be > 90% in some studies, except for the gray zone with a value of 0.86–0.93^16,30^. When the hyperemia zone was excluded, the accuracy of the correlation of RFR with FFR increased in the present study (93.1%) compared to that in other studies. In addition, we further analyzed the hyperemia zone by dividing it into positive (RFR: 0.86–0.89) and negative (RFR: 0.90–0.93) areas. Notably, the accuracy rate in the negative area was 87.7%, similar to that in the hyperemia-free zone, whereas the accuracy rate in the positive area was only 47.8%. Previous iFR studies suggested the need for a hybrid approach in the entire hyperemia zone because of the low accuracy rate^11,13,22^, and a recent RFR-FFR study also reported a low accuracy rate of 68% in the hyperemia zone (RFR: 0.86–0.93)^16^. However, because the present study showed that the accuracy rate in the negative area was similar to that in the hyperemia-free zone, we cautiously suggest that RFR alone could predict the functional significance of the intermediate lesion, except in the positive area of the hyperemia zone (13.8% of participants in the present study). The strategy of additionally implementing FFR in the positive area of the hyperemia zone might improve the diagnostic accuracy of RFR and can avoid unnecessary adenosine- or nicorandil-induced hyperemia to ensure patients’ comfort and time and cost savings. In the future, a large-scale randomized trial should be conducted using FFR and NHPR, including iFR with RFR, which can overcome the limitations of iFR and RFR to evaluate intermediate coronary artery stenosis accurately.

### Study limitations

This study has several limitations. First, it was a prospective observational study conducted in a single center. Second, among NHPR, iFR, which is the gold standard, could not be compared directly with RFR in this study. However, since RFR showed a strong correlation with Pd/Pa, it might be replaced by the iFR value. Third, the percentage of stenosis > 70% (26/474, 5.5%) was limited, which reduced the validity in reference to angiographically significant lesions (see Supplementary Fig. S2). To address these limitations, a large-scale multicenter trial using RFR with FFR is required.

## Conclusions

RFR and FFR values showed a good correlation with a high accuracy rate (RFR ≤ 0.89, FFR ≤ 0.8) in real-world clinical practice for intermediate coronary lesions. In addition, for further analysis of the hyperemia zone, RFR alone could evaluate the functional significance of coronary artery stenosis without unnecessary hyperemia, except in the positive area (RFR: 0.86–0.89) of the hyperemia zone.

### Supplementary Information

Supplementary Information.

## Data Availability

The datasets used and/or analysed during the current study available from the corresponding author on reasonable request.
