# Supplementary material for: Invasive physiologic assessment of coronary artery stenosis by resting full-cycle ratio and fractional flow reserve: a prospective observational study
Source: Sci Rep. 2023 Sep 22;13:15783. doi: 10.1038/s41598-023-43082-1 (PMC10516888; doi:10.1038/s41598-023-43082-1)
Supplement: Supplementary file 1 — Supplementary Information. [file 41598_2023_43082_MOESM1_ESM.docx]

**SUPPLEMENTARY MATERIAL**

**Invasive physiologic assessment of coronary artery stenosis by resting full-cycle ratio and fractional flow reserve: a prospective observational study**

Oh-Hyun Lee, MD^1†^, Ji Woong Roh, MD, PhD^1†^, Yongcheol Kim, MD, PhD^1*^, Seok-Jae Heo, PhD^2^, Eui Im, MD^1^, and Deok-Kyu Cho, MD^1*^

**CONTENTS**

Supplementary Figure S1. Histograms of the FFR and RFR values 2

Supplementary Figure S2. Box plots of the FFR and RFR values 3

Supplementary Table S1. Predictors of FFR and RFR discordance 4

**Supplementary Figure S1. Histograms of the fractional flow reserve (FFR) and resting full-cycle ratio (RFR) values.**


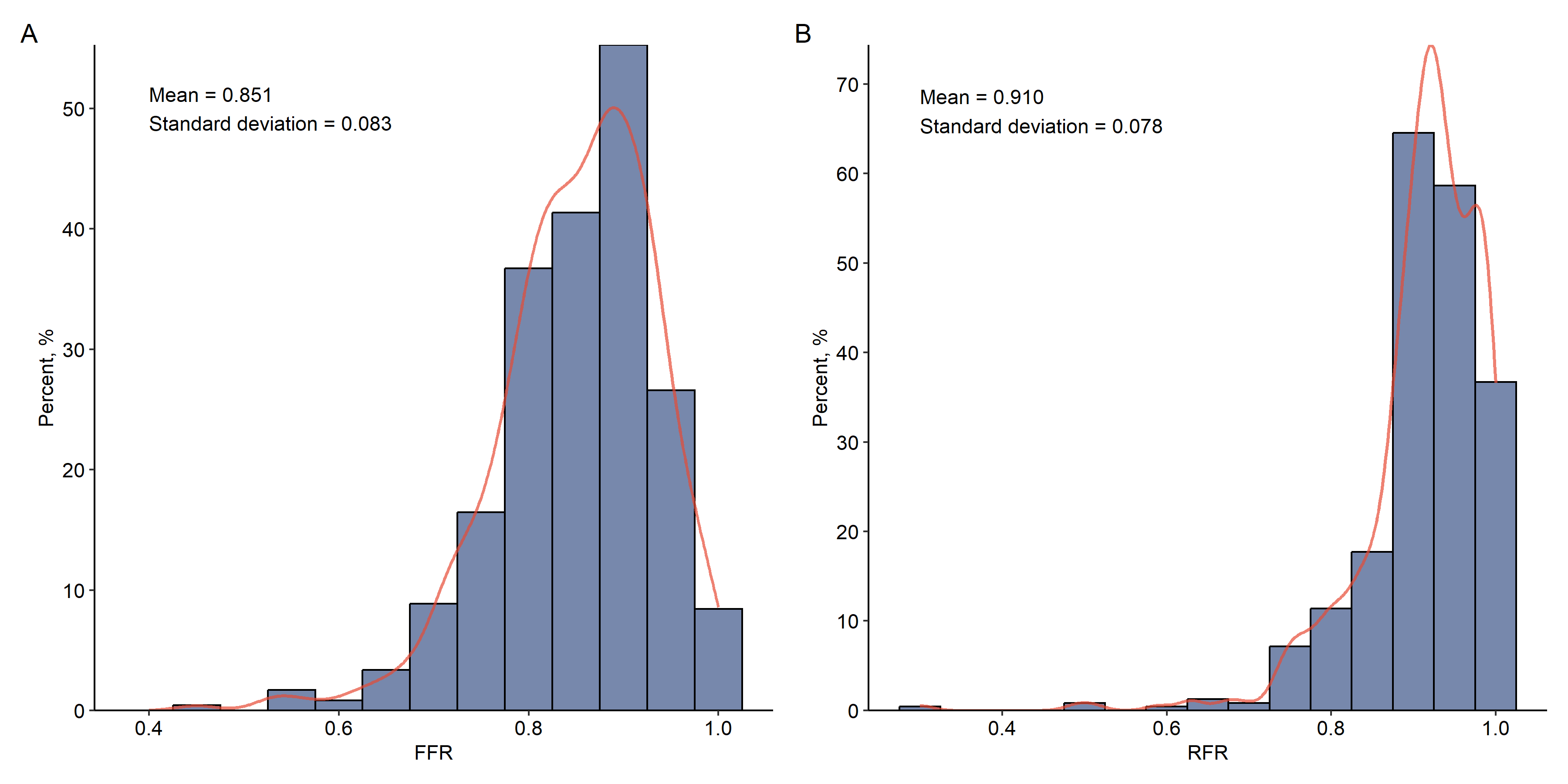


(A) FFR, (B) RFR

FFR, fractional flow reserve; RFR, resting full-cycle ratio.

**Supplementary Figure S2. Box plots of the FFR and RFR values.**


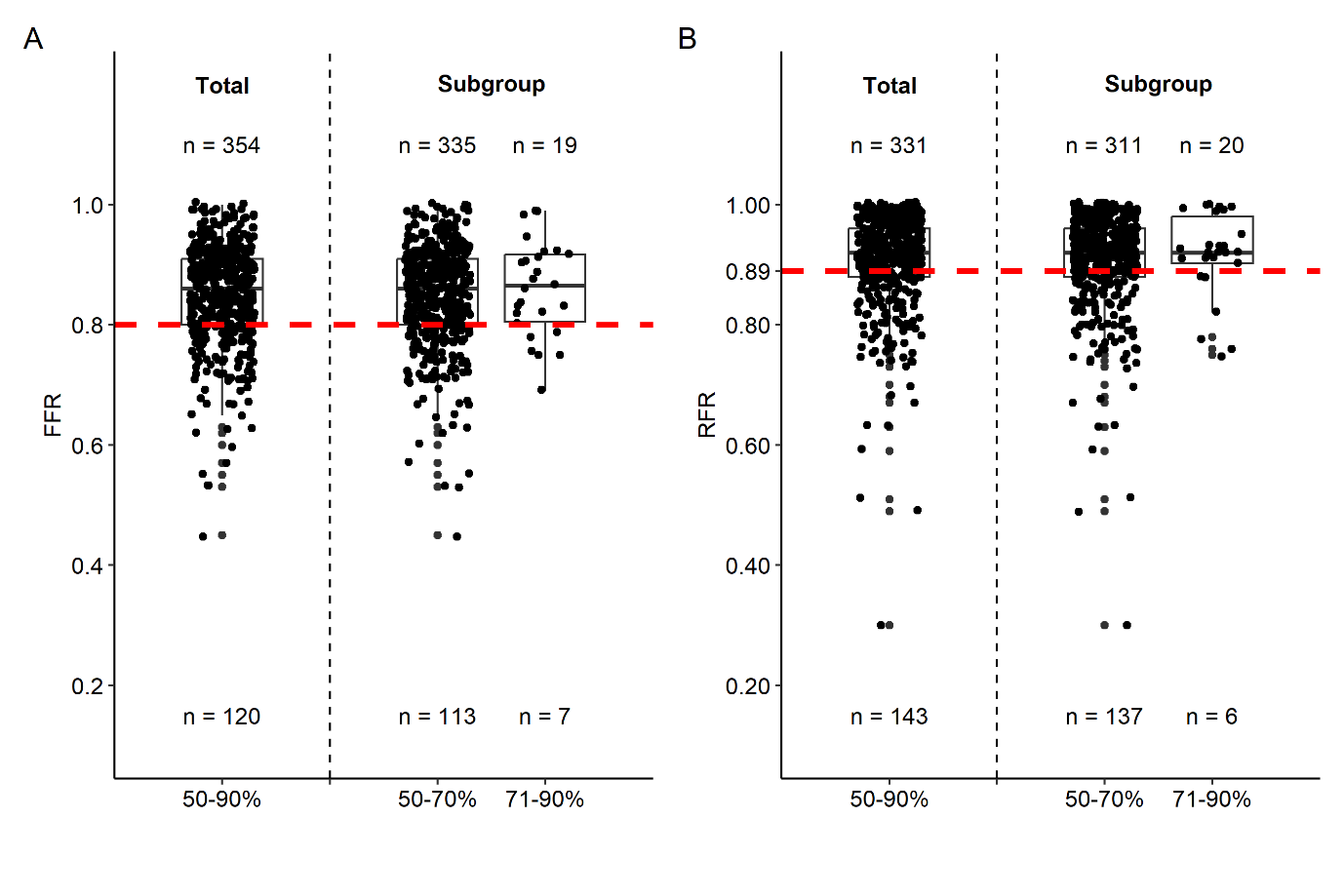


(A) FFR, (B) RFR

FFR, fractional flow reserve; RFR, resting full-cycle ratio.

**Supplementary TABLE S1. Predictors of FFR and RFR discordance**

| *Variables* | Univariate Analysis | | Multivariate Analysis | |
| --- | --- | --- | --- | --- |
|  | HR (95% CI) | P Value | HR (95% CI) | P Value |
| Age | 0.80 (0.48-1.33) | 0.393 |  |  |
| Female | 1.05 (0.60-1.79) | 0.871 |  |  |
| Hypertension | 0.86 (0.51-1.51) | 0.597 |  |  |
| Diabetes mellitus | 1.76 (1.06-2.96) | 0.032 | 1.80 (1.07-3.07) | 0.026 |
| Prior PCI | 1.00 (0.52-1.82) | 0.995 |  |  |
| Prior myocardial infarction | 1.28 (0.56-2.66) | 0.528 |  |  |
| Peripheral artery disease | 1.14 (0.17-4.44) | 0.868 |  |  |
| Acute myocardial infarction | 1.38 (0.57-2.98) | 0.441 |  |  |
| LAD lesion | 1.69 (0.98-3.04) | 0.067 | 1.74 (0.99-3.07) | 0.054 |
| Diffuse lesion | 1.10 (0.60-1.94) | 0.746 |  |  |

FFR, fractional flow reserve; RFR, resting full-cycle ratio; PCI, percutaneous coronary intervention; LAD, left anterior descending artery.
